# Predicting the Elastic Modulus of Recycled Concrete Considering Material Nonuniformity: Mesoscale Numerical Method

**DOI:** 10.3390/ma17020379

**Published:** 2024-01-12

**Authors:** Jing Zhang, Xuejun Zhu, Mingyuan Zhou, Xianwen Huang

**Affiliations:** 1 Department of Architecture and Engineering, Yancheng Polytechnic College, Yancheng 224005, China; 2 School of Civil Engineering, Jiangsu University of Science and Technology, Zhenjiang 212003, China; zmy15606102449@163.com; 3 School of Civil Engineering, Nantong University, Nantong 226019, China; 15062955958@163.com; 4 School of Civil Engineering, Suzhou University of Science and Technology, Suzhou 215009, China

**Keywords:** concrete, elastic modulus, numerical simulation, mesoscale, Monte Carlo

## Abstract

The evaluation of the elastic modulus of recycled concrete is one of the focuses of civil engineering and structural engineering, which is not only related to the stability of building structures but also related to the resource utilization of concrete. Therefore, based on the IRSM method in mesoscale, a novel model for predicting the elastic modulus of recycled concrete is proposed which has the advantages of being low-cost and high-precision, amongst others, compared to theoretical and experimental methods. Then, the influence of coarse aggregate, contact surface, gelling material, and air bubbles on the elastic modulus of recycled concrete is studied. The IRSM model includes four processes: Identification, Reconstruction, Simulation, and Monte Carlo, which can accurately reconstruct the geometric characteristics of coarse aggregate, efficiently reconstruct the coarse aggregate accumulation model, and quickly analyze the elastic modulus of concrete, as well as fully consider the nonuniform characteristics of coarse aggregate distribution and shape. Compared with the experimental results, the error is less than 5%, which verifies the rationality of the IRSM method. The results of the parametric analysis show that the influence of each factor on the elastic modulus of concrete in descending order is elastic modulus of cement, elastic modulus of coarse aggregate, content of coarse aggregate, content of air voids, elastic modulus of contacting surface, and thickness of contacting surface, and the corresponding Pearson’s Coefficients are 0.688, 0.427, 0.412, −0.269, 0.188, and −0.061, respectively, in which the content of air voids and thickness of contact surface have a negative effect on the elastic modulus of concrete. These influences mainly affect the deformation resistance (elastic modulus) of concrete through “force chain” adjustment, including the force transfer effect, number of paths, and integrity.

## 1. Introduction

As a kind of high-performance engineering material, concrete is widely used in construction, bridges, dams, and other man-made structures [1,2]. With the development of buildings towards higher, deeper, and larger volumes, higher performance is put forward for concrete materials, such as compressive strength, tensile strength, fatigue characteristics, and elastic modulus [3]. In addition, with the deterioration of the ecological environment, researchers are making efforts to build sustainability [4,5,6], such as using green building materials, low-carbon engineering designs, and efficient construction technology.

In terms of green building materials, the reuse of construction waste is a good method [7,8], which can not only reduce the processing cost of construction waste but also reduce the cost of building materials. Therefore, some scholars have put forward the research plan of recycled concrete (RC), which uses recycled construction waste to replace part of coarse aggregate [9]. In terms of material design, in order to find concrete materials that meet specific performance requirements, engineers generally use experimental research [10], numerical simulation [11], theoretical analysis [12], and empirical estimation [13]. Experimental research is the most reliable, but its high test and time cost makes it difficult for engineers to accept. Theoretical analysis and empirical estimation are two kinds of concrete design methods based on theoretical model and engineering experience, respectively, but their accuracy is based on the rich experience of engineers, which limits the wide application of such methods. Numerical simulation is a new way to predict concrete properties and has been widely used in civil engineering in recent years. Due to the greater discretization of recycled materials compared with traditional materials (from the source of construction waste, processing technology, powder content, etc.), these uncertainties make experimental research short in terms of economic cost and time cost, and theoretical and empirical formulas cannot accurately predict the elastic modulus of recycled concrete. Therefore, numerical solutions are a low-carbon way to solve these problems.

In order to ensure the rationality of the numerical simulation model, it is necessary to establish a refined concrete geometric model [14,15]. Abdelmoumen et al. [16] established a prediction model of the elastic modulus of particle-reinforced composites considering coarse aggregate, interfacial layer, and filler and proved the feasibility of numerical simulations in elastic modulus prediction. Weigger et al. [17] used disjoint random spheres to simulate the spatial distribution characteristics of coarse aggregates and controlled different particle gradations by radius. Huang et al. [18] constructed a particle stacking model considering particle shape and surface characteristics by means of feature recognition and reconstruction. Dehghanpoor et al. [19] used ellipsoid to simulate coarse aggregate and established a concrete coarse aggregate deposition model that could consider particle shape and orientation. Garboczi et al. [20,21] considered concrete coarse aggregate as a fixed shape (spherical, regular polygon, etc.) and studied the influence of aggregate shape, diameter, and interface defects on concrete strength. In the study of Zhou et al. [22], concrete aggregate is considered to be a two-dimensional polygon with irregular shape, and the influence of the strength of the contact surface between aggregate and cementing material is considered through the interface layer. Chen et al. [15,23] further considered the shape of coarse aggregate and proposed a mesoscale model construction method for concrete with an irregular shape of coarse aggregate. In the study of Shuguang et al. [24,25], a real mesoscale numerical model of concrete was established based on CT scan images, and the influence of the interface between aggregate and cementing material was fully considered through thickening interface elements.

Unlike traditional concrete composed of coarse aggregate, fine aggregate, and cementing material [26], recycled concrete may also contain recycled coarse aggregate [27] or air bubbles [28]. In addition, in order to further reduce the impact of concrete material production on the environment, some concrete will be mixed with an air-entraining agent, which can greatly improve the construction workability of concrete and ensure the quality of a construction. At the same time, the amount of cementing material per unit volume can be reduced, and the carbon emissions in the production process of concrete material can be reduced. In concrete structures, the performance of the contact surface between coarse aggregate and cementing material [29] has a huge impact on its overall performance, which is reflected in the aspects of compressive strength, failure mode, elastic modulus prediction, and so on. In recycled concrete, because recycled aggregate is broken by construction waste, the surface of coarse aggregate is rougher than that of traditional coarse aggregate, and its strength may be weaker than the contact surface between traditional concrete and cementable material, which brings challenges to the prediction of the mechanical properties of recycled concrete.

Based on the above analysis, in order to design recycled concrete materials that meet the elastic modulus requirements under the premise of low carbon, referred to as the IRSM (Identification—Reconstruction—Simulation—Monte Carlo) numerical analysis method in mesoscale, a novel elastic modulus prediction model was established considering the actual coarse aggregate topography, composition diversity (coarse aggregate, gelling material, bubbles, etc.), and nonuniformity of the component distribution of concrete. The influencing factors of the elastic modulus of recycled concrete are analyzed in order to guide the engineering application of high-performance recycled concrete.

## 2. Mesoscale Numerical Model

In order to establish an accurate prediction model of the elastic modulus of concrete, based on the research of Huang et al. [30], an IRSM (Identification—Reconstruction—Simulation—Monte Carlo) prediction method for the elastic modulus of concrete was proposed.

**Figure 1:**
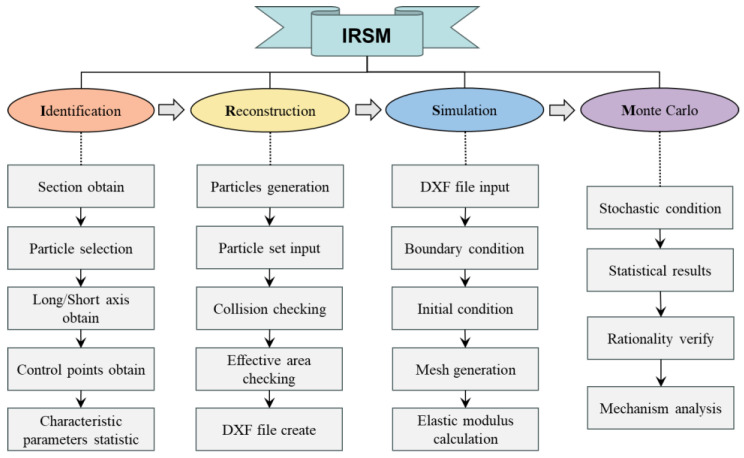
Introduction to IRSM methods.

As shown in Figure 1, the method mainly includes four processes, namely, Identification, Reconstruction, Simulation, and Monte Carlo calculation. For identification, the purpose is to extract the geometric features of coarse aggregates in concrete [31], such as particle size, long-axis size, roughness, etc. For the reconstruction process, the purpose is to reverse reconstruct the geometric characteristics of coarse aggregate in concrete based on the identified characteristic parameters and rationally put it into the set area. For the evaluation process, the purpose is to calculate the elastic modulus of concrete by numerical simulation based on the generated concrete elastic modulus calculation model, combined with the initial conditions of the model, attribute parameters, and boundary conditions. For the Monte Carlo method [32,33], the purpose is to fully consider the nonuniformity of the coarse aggregate placement position and geometric characteristics in concrete so as to obtain the predicted elastic modulus of concrete with statistical significance.

### 2.1. Identification for Concrete Model

According to Hohanson’s studies [34,35], it can be found that particle shape has great influence on the material mechanical properties and failure modulus. Therefore, in order to better reveal the influence of coarse aggregate on the elastic modulus of concrete, it is necessary to quantify the geometric features of coarse aggregate. Therefore, an efficient feature recognition method for concrete coarse aggregate is proposed, which can accurately identify the geometric size and angular characteristics of particles. The specific recognition process includes cross-section photography, coarse aggregate acquisition, long-axis acquisition, short-axis acquisition, feature point acquisition, and polar axis acquisition.

**Figure 2:**
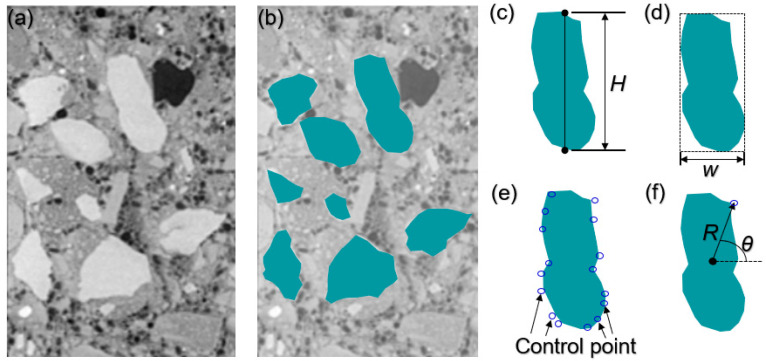
Characteristics identification of concrete coarse aggregate. (**a**) Concrete section; (**b**) Aggregate marked concrete; (**c**) The longest axis of coarse aggregate; (**d**) The width of of coarse aggregate; (**e**) The characteristic points of coarse aggregate; (**f**) The angle of of coarse aggregate.

Cross-section photography: The method of cross-section scanning, such as CT [36], is used to obtain nondestructive cross-section images of concrete. Cross-section images of concrete can also be obtained by means of first cutting and then optical photography, as shown in Figure 2a.

Coarse aggregate acquisition: The outline image of coarse aggregate particles is obtained based on pixel differences and imported into Matlab software, as shown in Figure 2b.

Long-axis acquisition: Based on Matlab software (2018), the longest axis of coarse aggregate is obtained by scanning boundary point data, and is defined as H, as shown in Figure 2c.

Short-axis acquisition: Based on the long axis of the identified particle, the long axis is extended along the vertical line to both ends until the particle is completely wrapped. The short side of the wrapped outer rectangle is the width W of the coarse aggregate, as shown in Figure 2d.

Feature point acquisition: According to Matlab software, the control point of coarse aggregate is extracted by setting the smooth threshold (the angle difference between tangent lines between adjacent coarse aggregate contour points), as shown in Figure 2e.

Polar axis acquisition: The midpoint of the outer rectangle is point A, the feature point is point B, the line segment is constructed, and the length R of the line segment and Angle θ between the line segment and horizontal line is measured.

**Figure 3:**
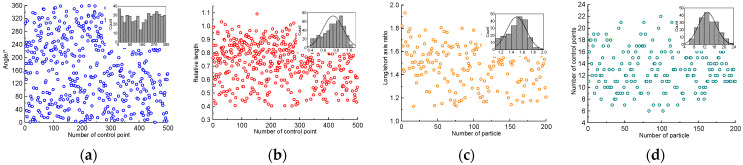
Identification results of feature parameters. (**a**) feature angles; (**b**) relative length; (**c**) long-axis/short-axis ratio; (**d**) the number of control points.

**Figure 4:**
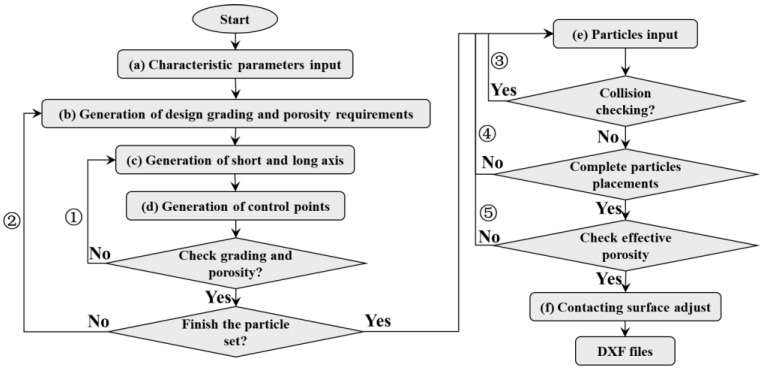
Reconstruction of coarse aggregate accumulation model.

In order to reconstruct the geometric model of concrete coarse aggregate, based on a set of CT scan results, the identification method proposed in this paper was adopted to identify and extract the geometric features of coarse aggregate, and the results are shown in Figure 3. Figure 3a shows the recognition results of concrete feature Angle θ. It can be found that the spatial distribution of the feature angle is significantly random and presents the characteristics of random distribution; Figure 3b shows the identification results of the relative length *α* of coarse aggregate, which ranges from 0.4 to 1.1, and the calculation formula is shown in Equation (1). Through the statistical analysis of relative length *α*, it can be found that the relative length of coarse aggregate presents a normal distribution trend in spatial distribution, and this trend will be applied to the reconstruction of coarse aggregate. Figure 3d shows the identification results of the long axial ratio *β* of coarse aggregate, which reflects the morphological characteristics of the particles, such as elongated, spherical, etc. The identification results show that the *β* distribution of coarse aggregate is in the range of 1.0~2.0 and presents a normal distribution. Figure 4d shows the identification results of the number of control points N in 200 particles, which reflects the number of feature points in a single particle and is an important parameter for later model reconstruction. The identification results show that the number of feature points in the coarse aggregate ranges from 4 to 23, and the average is 13, which indicates that there are normal characteristics in the statistical sense.

(1) $$\alpha =\frac{2R}{H}$$

(2) $$\beta =\frac{L}{W}$$

### 2.2. Reconstruction for Concrete Model

Based on Huang’s studies [30], a reconstruction method for the concrete to match the identified method proposed in this manuscript is put forward, and the corresponding construction process is shown in Figure 4. Firstly, the geometric parameters of coarse aggregate are input into the calculation model. Then, according to the grading and porosity requirements of coarse aggregate, the coarse aggregate content Ac in each grading range is calculated. Then, according to the grading requirements, the short-axis length *W* of coarse aggregate is generated, and the corresponding long-axis *H* of coarse aggregate is constructed according to the long-axis ratio *β*. Then, according to the number of control point parameters N, feature angle θ and relative length α are identified to reconstruct the corresponding coarse aggregate geometric contour. The constructed coarse aggregate is added to the coarse aggregate generation set, and whether the allowable error of the area value of a single gradation is satisfied is judged, as shown in the process ①. If it is not satisfied, the particle is re-generated; otherwise, it enters the next cycle. According to the gradation order from large to small, coarse aggregate generation sets within the corresponding gradation range are constructed successively, as shown in process ②, until all the generation sets are completed.

**Figure 5:**
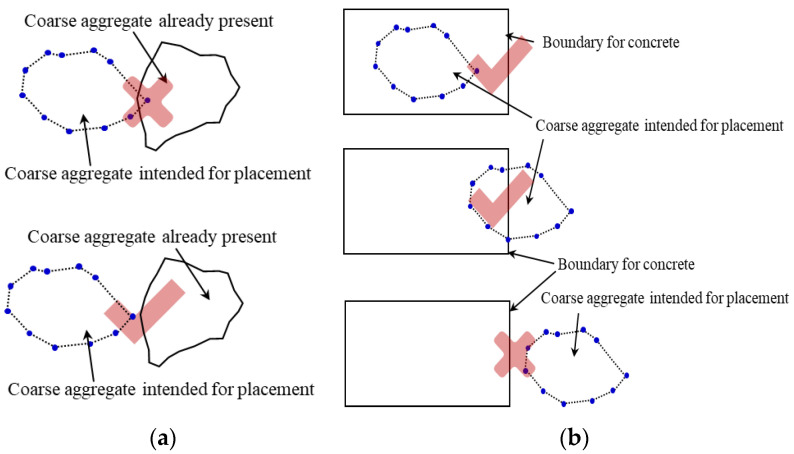
Checking of particle placement. (**a**) Coarse aggregates already present and intended for placement; (**b**) coarse aggregate and concrete boundary.

When the coarse aggregate generation set is constructed, the particles need to be put into the corresponding concrete space. The coarse aggregate is placed in the order from large to small so that a higher efficiency can be obtained [30]. When a new particle is placed, all the feature points of the new particle are tested against the particles that have already been placed. The new coarse aggregate will only be placed if all the feature points of the newly placed coarse aggregate are outside the already placed coarse aggregate as shown in Figure 5a (this is also known as the collision detection process). If some of the new coarse aggregate is inside the concrete space as shown in Figure 5b, this is repeated until all the pellets have been placed. Since part of the coarse aggregate may be outside the concrete space, which may cause the porosity of the concrete accumulation model to be different from the design porosity, it is necessary to conduct a secondary test to ensure that the generated porosity is within the allowable range of the design porosity error. For the consideration of the contact surface between coarse aggregate and cementing material, the coarse aggregate outline is mainly shifted inward, and the offset distance is equal to the thickness of the contact surface. Then all the coarse aggregate is put in and converted to a numerical output format that the software can recognize (DXF files are recommended).

### 2.3. Simulation for Concrete Model

**Figure 6:**
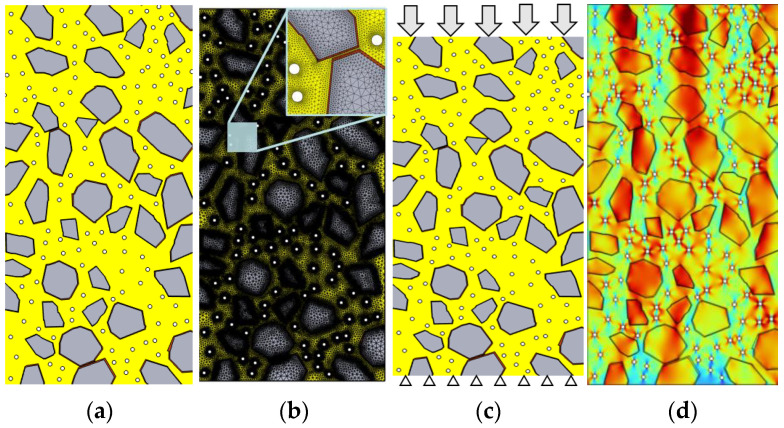
This is a figure. Simulation for the elastic modulus simulation of concrete. (**a**) Geometric model; (**b**) mesh division; (**c**) boundary conditions; (**d**) numerical results.

In order to predict the elastic modulus of concrete, based on the generated concrete geometric model containing contact surface, COMSOL software [37] was used to establish the corresponding numerical simulation model. The modeling and analysis process is shown in Figure 6. Figure 6a shows the geometric model in the numerical analysis, which mainly includes four parts, namely coarse aggregate, contact surface, bubble, and cementing material, all of which a have different elastic modulus. Figure 6b shows the results of the grid division of the calculated region. It should be noted that a smaller grid size, while allowing for more accurate prediction results, will increase the amount of computation. Therefore, the model adopts a “transitional” grid division method [38], that is, small-size grids are used for some small-size areas (such as contact areas and other areas of concern), while large-size grids are used for some large-size areas (such as coarse aggregate center and other cementing material areas), and a grid coefficient less than two times is used to amplify the two different sizes of the grids. The problem of nonconvergence that may exist in the calculation of the model is eliminated. Figure 6c shows the boundary conditions of the elastic modulus of concrete. In this model, fixed boundary conditions are set at the bottom of the concrete, boundary conditions of force *F* are set at the top, and free boundary conditions are set on both sides of the model. Figure 6d shows the stress distribution results of the model after compression. It can be seen from the Figure 6d that large stress distributions appear around the aggregate edges and bubbles, which is related to the stress concentration [11] caused by the elastic modulus of the material and geometric effects.

Based on the numerical analysis results, the deformation Δ*L* of concrete under rated load *F* is measured, and the elastic modulus of concrete is calculated by Equation (3).

(3) $$E=\frac{F\cdot b}{a\cdot \Delta L}$$

where *F* represents model load, N; Δ*L* represents the deformation of the model, m; *a* represents the model width, m; and *b* represents the height of the model, m.

### 2.4. Monte Carlo Consideration for Concrete Model

**Figure 7:**
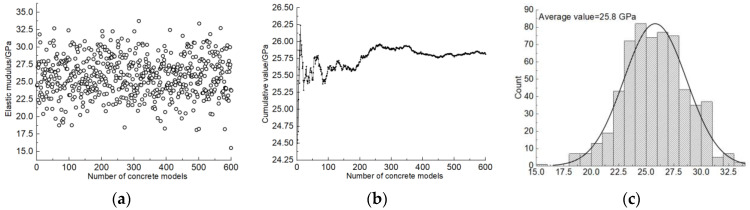
Monte Carlo results. (**a**) Calculation result statistics; (**b**) cumulative average; (**c**) result statistics.

In order to fully consider the nonuniform characteristics of the geometric shape and distribution characteristics of the concrete coarse aggregate, the Monte Carlo method was used for analysis. A set of typical analysis results are shown in Figure 7a. In Monte Carlo analysis, it is very important to determine a reasonable random number of times, which requires a balance between statistically significant results and model computations. Hence, the cumulative average of the calculation results under different calculation times was statistically analyzed, as shown in Figure 7b, and it can be found that the model is basically in a state of equilibrium when the calculation amount reaches 500 times, so 500 times is set as the standard calculation times of the model. The statistical results calculated by the model are shown in Figure 7c. It can be found that the calculated results of the concrete elastic modulus show a trend of normal distribution across the horizontal line, and the average value is 25.8 GPa.

## 3. Rationality Verification

**Figure 8:**
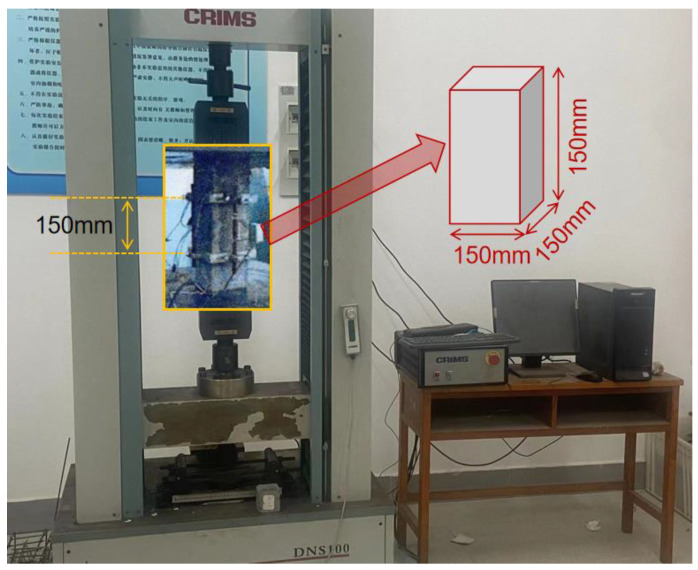
Experimental details for the elastic modulus of concrete.

In order to test and obtain the elastic model of concrete, prismatic samples with a length, width, and height of 150 mm, 150 mm, and 300 mm, consisting of C40 and C35 level concrete, were prepared (shown in Figure 8) based on the Standard for Testing Mechanical Properties of Ordinary Concrete (GB_T50081-2019) [39], and the deformation and load values of concrete samples were obtained by micro-deformation measuring the instrument and pressure module. It should be noted that the distance between the two marks is 150 mm, as shown in Figure 8.

Table 1 reflects the test results of elastic model quantities of concrete in two groups (six in each group). It can be found that the elastic modulus values of concrete in two tests are 32.2 GPa and 26.3 Gpa, respectively, and the corresponding numerical analysis predicted values are 33.5 Gpa and 25.8 Gpa, respectively; the difference between the two is 4.04% and 1.9%, respectively. The 5% error requirement of engineering design is satisfied, and the rationality of the numerical simulation model is proved. In addition, by comparing the values of the two, it can be found that the elastic modulus of the concrete mixed with recycled coarse aggregate is lower than that of traditional concrete, and the strain generated under the same pressure value is larger, which is mainly due to the poor mechanical properties of recycled coarse aggregate.

## 4. Parametric Analysis

The influencing factors of concrete elastic modulus mainly include coarse aggregate content, coarse aggregate properties, contact surface thickness, contact surface properties, cementing material type, and bubble content. Therefore, based on the IRSM method for predicting the elastic modulus of concrete proposed in this project, the elastic modulus of concrete under the influence of various variables is analyzed to better support the application of concrete engineering. Coarse aggregate is an important component of concrete structures. According to the results of the CT scan in this project and the research results of Liu et al. [40], the effective range of coarse aggregate is set at 20~50%. Considering that concrete coarse aggregate includes recycled coarse aggregate prepared by construction waste, the elastic modulus of the coarse aggregate is 20~50 Gpa. Based on CT test results and previous studies [11], the thickness of the contact layer between coarse aggregate and cementing material was set to 0~5 mm, and the elastic modulus was considered to be 10~30 Gpa. According to the test results of the elastic modulus of mortar and the research results of Chen et al. [23], the elastic modulus of cementing material is set at 5~30 Gpa. According to the amount of initiator and CT test results, the bubble content in concrete is set to 0~10%. According to the effective range of the above variables, parametric analysis was carried out, and the test scheme is shown in Table 2.

### 4.1. The Influence of Coarse Aggregate

**Figure 9:**
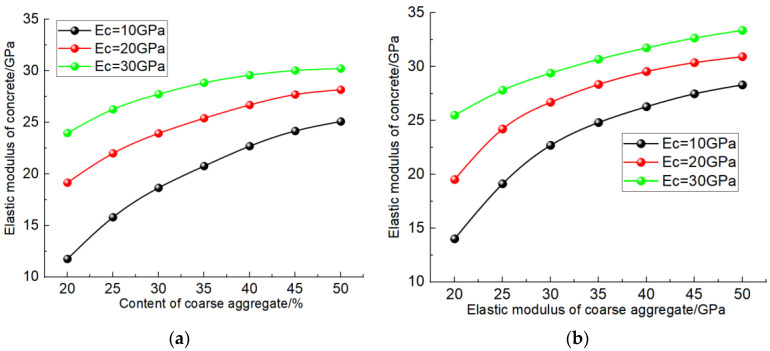
Simulation results considering the influence of coarse aggregate. (**a**) Content of coarse aggregate; (**b**) elastic modulus of coarse aggregate.

Figure 9 shows the simulation results at the different contents and elastic modulus of coarse aggregate. It can be seen from Figure 9a that with the increase in the coarse aggregate content, the elastic modulus of concrete presents a nonlinear increase, and the increasing trend gradually slows down. In the analysis of the reasons, with the increase in the coarse aggregate content, concrete gradually formed a mechanical skeleton composed of coarse aggregate, improved the deformation resistance of concrete, and thus showed a higher elastic modulus. When the coarse aggregate content reaches 40%, the mechanical skeleton inside the concrete has been perfected. Therefore, with the continuous increase in the mixed coarse aggregate content, the contribution of the increased coarse aggregate content to the mechanical skeleton gradually weakens, and the increase rate gradually slows down. Comparing the test results of different cementing materials, it can be found that the lower the elastic modulus of cementing materials, the greater the influence of the increase in the coarse aggregate content on the elastic modulus of concrete.

As shown in Figure 9b, under the premise of the same coarse aggregate content, with the increase in the elastic modulus of coarse aggregate, the predicted result of the elastic modulus of the concrete also gradually increases, showing a nonlinear growth trend. When the content of coarse aggregate is 40%, the force transfer skeleton composed of coarse aggregate inside concrete is relatively perfect, so the greater the elastic modulus of coarse aggregate, the stronger the deformation resistance of concrete, and the higher the elastic modulus. When the elastic modulus of cementing material is 10 GPa, the nonlinear trend of the elastic modulus of concrete increasing with the increase in the elastic modulus of coarse aggregate is obvious. When the elastic modulus of cementing material is 30 Gpa, the elastic modulus of concrete increases linearly with the elastic modulus of coarse aggregate. Analyzing the reasons for this, the lower elastic modulus of the cementified material limits the increase in the elastic modulus of the coarse aggregate on the deformation resistance of concrete, and the greater the difference between the elastic modulus of the coarse aggregate and the cementified material, the more obvious this limitation is. Therefore, with the increase in the elastic modulus of the coarse aggregate, the concrete containing a low elastic modulus of the cementified material shows a smaller increase in the elastic modulus.

### 4.2. The Influence of Contact Surface

**Figure 10:**
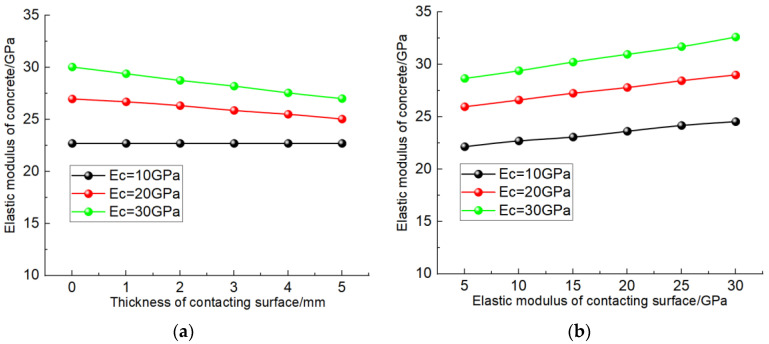
Simulation results considering the influence of thickness and elastic modulus of contact surface. (**a**) Thickness of contact surface; (**b**) elastic modulus of contact surface.

Figure 10 shows the simulation results at a different thickness and elastic modulus of contact surface between coarse aggregate and cementing material. As shown in Figure 10a, when the elastic modulus of the contact layer is the same as that of the cementing material (10 Gpa), the elastic modulus of concrete does not increase with the increase in the thickness of the contact layer. When the elastic modulus of the contact layer is less than the strength of the cementing material, the elastic modulus of the concrete gradually decreases with the increase in the thickness of the contact layer, and the greater the difference between the elastic modulus of the contact layer and the cementing material, the more obvious the reduction amplitude. Analyzing the reasons for this, the lower elastic modulus of the contact layer weakens the force transfer effect of the “force chain” of the coarse aggregate, and the greater the thickness of the contact layer, the larger the proportion of the contact layer in the “force chain”, the more obvious the weakening effect on the deformation resistance of the concrete, so the elastic modulus of the concrete increases with the increase in the thickness of the contact surface.

As shown in Figure 10b, the elastic modulus of concrete presents a linear growth trend with the increase in the elastic modulus of the contact surface, and the higher the elastic modulus of the cementing material, the more obvious the increase. The reason for this is that with the increase in the elastic modulus of the contact layer, the less the weakening effect of the force chain composed of coarse aggregate inside the concrete, the better the deformation resistance of the concrete, that is, the greater the elastic modulus. When the elastic modulus of the cementitious material is high, the contact layer has a greater influence on the “force chain” of the concrete, so the elastic modulus of the contact layer shows a greater influence on the cementitious material concrete with a high elastic modulus.

### 4.3. The Influence of Bubble

**Figure 11:**
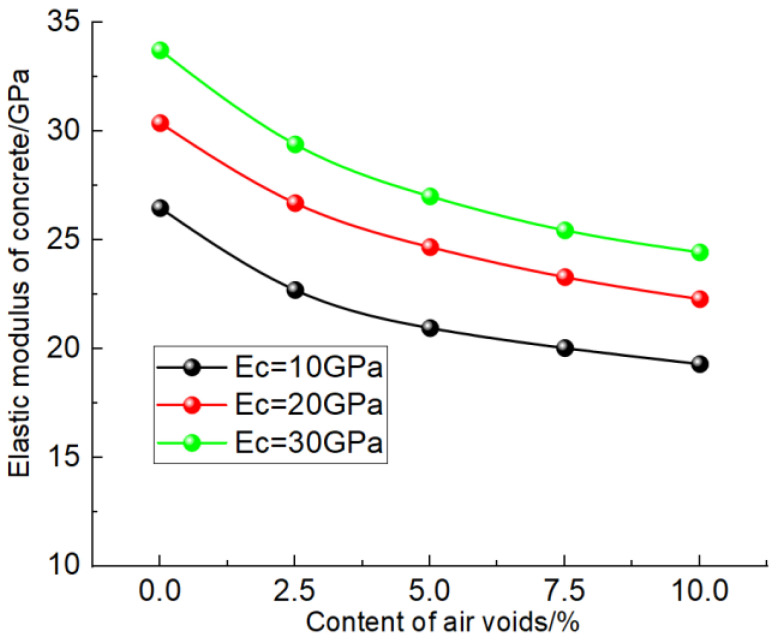
Simulation results considering the influence of bubble.

Figure 11 shows the evolution law of the concrete elastic modulus under different bubble contents. It can be seen from the Figure 11 that with the increase in the bubble content, the elastic modulus of concrete gradually decreases and presents a nonlinear trend. Analyzing the reasons for this, with the increase in the content of bubbles inside the concrete, the “force chain” will be affected by different degrees, such as disconnection, zigzag, etc., which will weaken the antideformation ability of the concrete, so the elastic modulus gradually decreases. When the bubble content is less than 5%, the effect on the “force chain” is significant, so the decrease amplitude of the elastic modulus of concrete is large. When the bubble content is greater than 5%, the “force chain” has been greatly disturbed, and the increase in bubbles has little impact on the “force chain”, so the decline gradually slows down. By comparing the test results of cementified materials under different elastic moduli, it can be found that the higher the elastic moduli of cementified materials, the greater the reduction caused by bubbles, which is related to the greater difference between the elastic moduli of bubbles and cementified materials. The greater the difference between the two, the more obvious the weakening effect of bubbles, and the greater the reduction in the elastic moduli of concrete.

### 4.4. Discussion and Analysis

**Figure 12:**
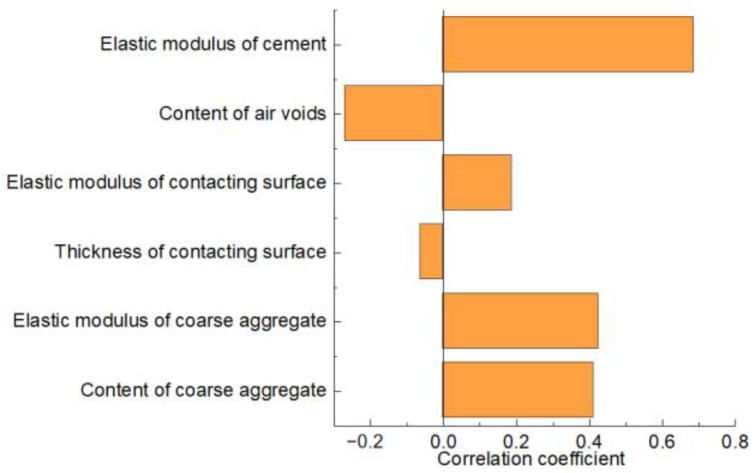
Simulation results for correlation analysis.

It is very important for the design and application of high-performance concrete to understand the influence of various factors on the elastic modulus of concrete. To this end, Pearson correlation analysis [41,42] was used for research, and the analysis results are shown in Figure 12.

Figure 12 shows the simulation results of the correlation coefficient, and it can be found that the influence factors on the elastic modulus of concrete in descending order is: elastic modulus of cement, elastic modulus of coarse aggregate, content of coarse aggregate, content of air voids, elastic modulus of contacting surface, and thickness of contacting surface, and the corresponding correlation coefficients are 0.688, 0.427, 0.412, −0.269, 0.188, and −0.061, respectively, in which the content of air voids and thickness of contacting surface have negative effects on the elastic modulus improvement in concrete. According to the results of the Pilsch analysis, it can be found that the elastic modulus and content of coarse aggregate and the performance of cementing material have the greatest influence on the elastic modulus of concrete. When construction waste is used to replace coarse aggregate or part of fine aggregate in concrete, attention should be paid to the weakening effect on the elastic modulus of concrete.

Through the analysis of the stress nephogram after concrete compression, it can be found that the influence of various influencing factors on the elastic modulus of concrete is mainly reflected in the force transfer effect, the number of paths, and the integrity of the “force chain”. The increase in the elastic modulus between coarse aggregate and contact surface will improve the force transfer effect of the “force chain”. The increase in the coarse aggregate content and the improvement in cementitious material properties will increase the force transfer path and improve the deformation resistance of concrete. The increase in the bubble and contact surface thickness will destroy the force transfer path and weaken the deformation resistance of concrete.

In addition, it should also be observed that the cementing material consisted of cement and fine aggregate, which were considered to be uniform materials in the manuscript. However, it is nonuniform and influences the mechanical properties of concrete. Hence, the method proposed in the manuscript is suitable for concrete, whose strength class is smaller than the C60 level, and more studies will be conducted in the future.

## 5. Conclusions

In order to accurately predict the elastic modulus of recycled concrete by means of numerical analysis, an IRSM method considering the geometric characteristics and nonuniformity of the distribution of concrete was proposed, and the rationality of the method was verified by comparison experiments, and parametric analysis was carried out based on the main components of concrete. The main conclusions are as follows:

(1) An efficient identification and reconstruction method for the geometric characteristics of concrete coarse aggregate is proposed, which can efficiently identify the long-axis, short-axis, feature point, and feature length of coarse aggregate and accurately construct the geometric model of the coarse aggregate with original structural characteristics according to these parameters.
(2) A concrete elastic modulus prediction IRSM model is proposed, which can consider the influence of coarse aggregate, contact surface, cementified material, bubbles, and other factors, and the rationality of the model is verified through experimental research (relative error is less than 5%).
(3) According to Pearson’s analysis, the influences of each influencing factor on the elastic modulus of concrete are as follows: elastic modulus of cement, elastic modulus of coarse aggregate, content of coarse aggregate, content of air voids, elastic modulus of contacting surface, and thickness of contacting surface, and the corresponding correlation coefficients are 0.688, 0.427, 0.412, −0.269, 0.188, and −0.061, respectively.
(4) It is revealed that the influence of various influencing factors on the elastic modulus of concrete is mainly reflected in the force transfer effect, the number of paths, and the integrity of the “force chain”. The increase in elastic modulus between coarse aggregate and the contact surface will improve the force transfer effect of the “force chain”. The increase in coarse aggregate content and the improvement in cementitious material properties will increase the force transfer path and improve the deformation resistance of concrete. The increase in bubble and contact surface thickness will destroy the force transfer path and weaken the deformation resistance of concrete.

## Figures and Tables

**Table 1:** Elastic modus obtained by experimental and numerical method.

No.	Exp-1 /GPa	Exp-2 /GPa	Exp-3 /GPa	Exp-4 /GPa	Exp-5 /GPa	Exp-6 /GPa	Exp-Ave /Gpa	Num /Gpa	Error
1	32.2	30.5	31.3	33.2	33.9	32.1	32.2	33.5	4.04%
2	26.1	27.9	24.9	25.3	26.3	27.	26.3	25.8	1.90%

**Table 2:** Analysis of influencing factors of elastic modulus of concrete.

Materials	Factors	Range
Coarse aggregate	Content/%	20, 25, 30, 35, 40, 45, 50
	Elastic modulus/Gpa	20, 25, 30, 35, 40, 45, 50
Contact surface	Thickness/mm	0, 1, 2, 3, 4, 5
	Elastic modulus/Gpa	5, 10, 15, 20, 25, 30
Bubble	Content/%	0, 2.5, 5, 7.5, 10
Cementing material	Elastic modulus/Gpa	10, 20, 30

## Data Availability

Data are contained within the article.
